# Supplementary material for: Is the Atomic Quadrupole Moment of a Carbon Atom in Graphene Zero? The Case for a Rational Definition of the Properties of Atoms in a Molecule
Source: J Phys Chem Lett. 2026 Jan 13;17(4):1000–8. doi: 10.1021/acs.jpclett.5c03649 (PMC12862809; doi:10.1021/acs.jpclett.5c03649)
Supplement: Supplementary file 1 [file jz5c03649_si_002.pdf]

Supporting Information: Supporting Information for "Is the atomic quadrupole moment of a carbon in graphene zero?: The case for a rational definition of the properties of atoms in a molecule."

Devin M. Mulvey,[†,‡] Kenneth D. Jordan,[*,‡] and Alston J. Misquitta[¶]

†Department of Chemistry, St. Bonaventure University, St Bonaventure, New York, 14778, United States of America

‡Department of Chemistry, University of Pittsburgh, Pittsburgh, Pennsylvania, 15260, United States of America

¶Department of Physics and Astronomy, Queen Mary University of London, London E1 4NS, United Kingdom

E-mail: jordan@pitt.edu

Contents

1 Change of Basis and Traceless Multipole Convention **S-3**

2 Methods **S-5**

2.1 Calculating GDMA and BS-ISA Multipoles S-6

2.2 Electrostatic Interaction Scans S-10

2.3 Calculating the Primitive Cartesian zz component of Graphene's Carbon in Periodic Boundary Conditions S-15

3 Additional Results **S-16**

3.1 Tables of Average Multipole Moments on Symmetry Equivalent Atoms S-17

3.2 Charge Oscillations S-22

3.3 Derivation of Electrostatic Interaction of a Point Charge with a Finite Disk of Quadrupolar and Edge Dipolar Density S-23

3.3.1 Charge Interacting with Plane of Quadrupolar Density S-24

3.3.2 Charge Interacting with Edge Ring of Dipolar Density S-27

3.3.3 Charge Interacting with Plane of Quadrupolar Density Terminated by a Ring of Dipolar Density S-31

References **S-31**

For those interested in reproducing this work we include example inputs for the calculations at the GitHub repo: https://github.com/dev-m-mulvey/quadrupole.git. In addition, we include the molecular coordinates for the PAHs as xyz files and the scripts used for plotting the results as Jupyter Notebooks.[S1]

1 Change of Basis and Traceless Multipole Convention

In the manuscript, we compared the results of our BS-ISA calculations to that of periodic boundary condition calculations. To do achieve this, we had to convert the 20-component of the traceless spherical harmonic quadrupole (Q_{20}) to it's primitive Cartesian counterpart ($\langle zz \rangle$). The manuscript did not describe this procedure, but we shall do so here to.

This conversion boils down to a few simple definitions and an algebraic rearrangement. To understand what the trace of a quadrupole moment tensor is and what it means for a moment to be traceless or primitive, we will start by showing the primitive Cartesian quadrupole tensor in matrix form:

$$\langle ij \rangle = \begin{bmatrix} \langle xx \rangle & \langle xy \rangle & \langle xz \rangle \\ \langle yx \rangle & \langle yy \rangle & \langle yz \rangle \\ \langle zx \rangle & \langle zy \rangle & \langle zz \rangle \end{bmatrix} \tag{1}$$

In the above definition, the primitive quadrupole tensor is a 3×3 matrix of nine pairs of Cartesian components i and j. The primary feature of interest is that the trace (sum of the diagonal) of the matrix can be any real number (i.e. $\mathrm{Tr}[\langle ij \rangle] = \langle xx \rangle + \langle yy \rangle + \langle zz \rangle \in \mathbb{R}$).

The traceless condition requires that the trace of the Cartesian quadrupole moment tensor vanishes, like so:

$$\mathrm{Tr}[\langle ij \rangle] = \langle xx \rangle + \langle yy \rangle + \langle zz \rangle \overset{!}{=} 0 \tag{2}$$

There are slight variations in the definitions used for traceless Cartesian multipole moments, however they all require the sum of the diagonal is zero. We use the traceless convention of Buckingham[S2,S3] on all multipoles above the dipole. For the traceless Cartesian quadrupole moment (Q_{ij}) specifically, Buckingham's traceless condition is defined as such,

$$Q_{ij} = \frac{3\langle ij \rangle - \mathrm{Tr}[\langle ij \rangle]\delta_{ij}}{2} \tag{3}$$

where all terms have been defined except δ_{ij}. The factor δ_{ij} is the Kronecker delta,

$$\delta_{ij} = \begin{cases} 0 & \text{if } i \neq j \\ 1 & \text{if } i = j \end{cases} \tag{4}$$

which ensures that the trace is only subtracted from the diagonal of $\langle ij \rangle$. Putting equation 3 in matrix form we have the following,

$$Q_{ij} = \begin{bmatrix} \frac{3\langle xx\rangle - (\langle xx\rangle + \langle yy\rangle + \langle zz\rangle)}{2} & \frac{3\langle xy\rangle}{2} & \frac{3\langle xz\rangle}{2} \\ \frac{3\langle yx\rangle}{2} & \frac{3\langle yy\rangle - (\langle xx\rangle + \langle yy\rangle + \langle zz\rangle)}{2} & \frac{3\langle yz\rangle}{2} \\ \frac{3\langle zx\rangle}{2} & \frac{3\langle zy\rangle}{2} & \frac{3\langle zz\rangle - (\langle xx\rangle + \langle yy\rangle + \langle zz\rangle)}{2} \end{bmatrix} \tag{5}$$

where we have substituted in the Cartesian components, expanded $\mathrm{Tr}[\langle ij \rangle]$, and distributed the factors of 3 and 1/2. One can see that $\mathrm{Tr}[Q_{ij}] = \sum_i Q_{ii} = 0$, hence the name, "traceless." Equation 3 generalizes to higher order multipoles (e.g. for the traceless octupole $\mathrm{Tr}[Q_{ijk}] = \sum_i Q_{iii} = 0$) and we refer readers who are interested in seeing more to the relevant references.[S2,S3]

An equivalent definition of equation 3 (and the one we employ in our change of bases) is,

$$Q_{ij} = \frac{3\langle ij \rangle - \langle\langle r^2 \rangle\rangle \delta_{ij}}{2} \tag{6}$$

where $\langle\langle r^2 \rangle\rangle$ is the spherical average of electronic density weighted by the square of its radial extent, $\int \rho(\mathbf{r}) r^2 d\mathbf{r}$, which is equivalent to $\mathrm{Tr}[\langle ij \rangle]$. This alternate definition is necessary for the aforementioned change of basis, as the distributed multipoles of a method like BS-ISA are traceless and in a spherical harmonic basis, but the output does provide $\langle\langle r^2 \rangle\rangle$ for every atom in the molecule.

With this information the aforementioned conversion from Q_{20} to $\langle zz \rangle$ starts with a

rearrangement of equation 6,

$$\langle ij \rangle = \frac{2Q_{ij}}{3} + \frac{\langle\langle r^2 \rangle\rangle \delta_{ij}}{3} \tag{7}$$

where all components in equation 7 are in the Cartesian basis, thus $i = j = z$ and:

$$\langle zz \rangle = \frac{2Q_{zz}}{3} + \frac{\langle\langle r^2 \rangle\rangle}{3} \tag{8}$$

Now all that remains is a substitution of the traceless of the traceless Cartesian Q_{zz} with its spherical harmonic counterpart Q_{20}. The spherical harmonic basis employed by BS-ISA is the same as that defined by Stone and if one refers to Appendix E of the book "The Theory of Intermolecular Forces", they will find that $Q_{zz} = Q_{20}$.[S4] Thus, we arrive at the following conversion for traceless spherical harmonic to primitve Cartesian quadrupole component,

$$\langle zz \rangle = \frac{2Q_{20}}{3} + \frac{\langle\langle r^2 \rangle\rangle}{3} \tag{9}$$

where all terms have been previously defined.

2 Methods

In this section we describe:

- The methods used to calculate BS-ISA and GDMA atomic multipoles.
- The method of constructing the $\mathrm{Q}_{20}^{\mathrm{C}} + \mathrm{Q}_{1\mathrm{m}}^{\mathrm{CH}}$ electrostatic model.
- The processes used for calculating the long and short range (charge penetration) electrostatic interaction error scans.
- The procedure used to calculate the primitive $\langle\langle zz \rangle\rangle$ component of the quadrupole on a carbon atom in graphene via periodic boundary condition calculations.

2.1 Calculating GDMA and BS-ISA Multipoles

BS-ISA[S5,S6] and GDMA[S7–S9] multipoles reported in this work were calculated using the following process:

1. We asymptotically correct the exchange-correlation (XC) potential of PBE0.[S10,S11] This requires knowing both the first ionization potential (IP) and energy of the highest occupied molecular orbital (HOMO). First, we calculate the IP of the molecule using the domain-based local pair natural orbital (DLPNO) version of equation of motion (EOM) coupled cluster theory with single and double excitations (CCSD) for ionization potentials (IP-EOM-DLPNO-CCSD)[S12,S13] as implemented in ORCA v.4.2.0.[S14]

    (a) A restricted Hartree-Fock (RHF)[S15–S17] reference wavefunction is used for IP-EOM-DLPNO-CCSD. The orbital basis used is cc-pVTZ[S18] and the `verytightscf` command is used to set the convergence criteria. For $C_{96}H_{24}$, we employ the RIJ-COSX approximation where the Coulomb integrals are density fit (RI-J) and the exchange integrals are estimated semi-numerically via the, "chain-of-spheres," approximation (COSX).[S19,S20] For density fitting the Coulomb integrals we employ the def2-universal Coulomb fitting basis.[S21]

    (b) To reduce the cost of obtaining the IP, IP-EOM-DLPNO-CCSD uses DLPNO to transform the integrals of CCSD from their canonical molecular orbital form to local pair natural orbitals (LPNOs), which essentially reduces the number of occupied and virtual orbitals that are used in the CCSD and EOM calculations.[S14] We use the `normalPNO` setting in ORCA, but reduce by an order of magnitude the threshold that determines the pairs to be treated via CCSD `TCutPairs`$=1e^{-5}$. We found that these settings afford a reasonable compromise between accuracy and expense for $C_{96}H_{24}$. In performing the DLPNO transformation the resolution of identity (RI) approximation is employed[S22] using the cc-pVTZ-RI fitting basis.[S23]

2. Next, we calculate the HOMO energy of the molecule using the density fitted PBE0

implemented in Psi4 v.1.4a2.dev213[S24–S26] in the basis that will be used to calculate the multipole moments. The density fitted SCF in this step and the following step employ Coulomb-exchange (JK) fitting bases.

3. Calculate the multipole moments using a development version of CamCASP v.7.2.2[S5,S6] interfaced to Psi4 v.1.4a2.dev213.

   (a) The IP and HOMO energy determined in the two previous steps are used to asymptotically correct the the XC potential of PBE0 via the gradient regulated asymptotic correction (GRAC)[S27] method implemented in Psi4. The PBE0(AC) calculations used an energy convergence cutoff of `e_convergence` $= 1 \times 10^{-11}$ a.u. and integrals with a value of `ints_tolerance` $< 1 \times 10^{-10}$ were dropped. Otherwise, the default convergence/screening settings of Psi4 were used. The resulting molecular orbitals and electronic density were passed to CamCASP as a formatted checkpoint file to determine BS-ISA and GDMA multipoles.

   (b) For GDMA, the multipoles were determined directly from the density matrix and orbitals from the Psi4 calculation. Stone's 2005 GDMA algorithm[S9] was used with "Grid Smoothing 3". This parameter governs the weights of points in the Becke grids for each atom. The real-space partitioning algorithm was used for all primitive basis functions with exponent greater than 3.0.

   (c) BS-ISA is a hybrid real-basis space extension of Lillestolen and Wheatley's ISA method.[S28] In BS-ISA, all the quantities that appear in the Stockholder equations (molecular density, atomic densities, and shape functions) are expanded in auxiliary bases. In this work, the atomic densities are expanded in RI fitting basis sets that have the s-block replaced with the ISA set2 s-functions developed by Misquitta, Stone, and Fazeli.[S5] The shape functions, which are also referred to as pro-atomic densities in the literature, are fit using only the ISA set2 s-function basis. The results reported in the main document make use of a custom basis

we developed for fitting the molecular density. However, we did try fitting the molecular density with RI fitting basis sets and the ISA set2 s-function basis as well. The combinations of orbital and auxiliary bases we tested are shown in Table S1 and the details of why we developed a new fitting basis for the molecular density are in the text following the table. We use the, "ISA-A," algorithm to partition the molecular density into atomic domains and the atomic multipole moments are calculated using real-space grids (referred to as ISA-GRID moments in the literature[S6]). Adjustments were made to the real-space grids and algorithmic convergence parameters of this method. The details of these refinements will be discussed in an upcoming publication by the developers of the CamCASP package. We encourage the reader to review the relevant citations[S5,S6] and look at the example input we provide in the GitHub repo, which contains the custom basis we developed as well.

A variety of orbital and fitting basis set combinations were tested in steps 2 and 3 of the above list to assess the sensitivity of the atomic multipoles. In Table S1 we tabulate all combinations.

Table S1: Various bases tested in the density fitted SCF calculation and calculation of atomic multipole moments.

Shorthand	Orbital	SCF-Auxiliary[a]	Molecular-Auxiliary[b]	Atom-Auxiliary[c]
aDZ/aDZ/aDZ[d]	aug-cc-pVDZ	aug-cc-pVDZ-JKfit	aug-cc-pVDZ-RIfit + set2[e]	aug-cc-pVDZ-RIfit + set2
aDZ/aDZ/aTZ	aug-cc-pVDZ	aug-cc-pVDZ-JKfit	aug-cc-pVDZ-RIfit + set2	aug-cc-pVTZ-RIfit + set2
aTZ/aTZ/aTZ	aug-cc-pVTZ	aug-cc-pVTZ-JKfit	aug-cc-pVTZ-RIfit + set2	aug-cc-pVTZ-RIfit + set2
aTZ/aTZ/aQZ	aug-cc-pVTZ	aug-cc-pVTZ-JKfit	aug-cc-pVTZ-RIfit + set2	aug-cc-pVQZ-RIfit + set2
aTZ/Pitt-fit/aQZ	aug-cc-pVTZ	aug-cc-pVTZ-JKfit	Pitt-fit[f]	aug-cc-pVQZ-RIfit + set2

[a] Basis used to density fit the SCF calculation.
[b] Basis used to expand the molecular density in BS-ISA algorithm.
[c] Basis used to expand the AIM densities are expanded in BS-ISA.
[d] Shorthand designation of the basis sets used in this text.
[e] + set2 indicates that the ISA set2 basis replaces the s-block of the RIfit basis.
[f] A custom basis we developed. See text for more information.

The orbital bases are of the aug-cc-pVXZ family,[S18,S29,S30] the SCF-Auxiliary bases used for density fitting are of the cc-pVXZ Coulomb-exchange fitting (-JK) family with diffuse

functions added.[S31] Note that the short hand basis notation in Table S1 applies only to BS-ISA as it includes the auxiliary basis sets. Only the orbital basis is used in GDMA, so only one entry will appear in its short hand (e.g. The aTZ/aTZ/aTZ, aTZ/aTZ/aQZ, and aTZ/Pitt-fit/aQZ calculations for BS-ISA will all be notated as aTZ for GDMA.)

As noted previously, BS-ISA makes use of extensive density fitting. Initially, we fit both the molecular PBE0(AC) density ($\rho(\mathbf{r})$) and atomic densities $\rho^a(\mathbf{r})$ in CamCASP with the aug-cc-pVXZ-RI bases[S23] with their s-block replaced by the ISA set2 s-function basis.[S5] However, the model ESP of the BS-ISA moments at the aTZ/aTZ/aQZ level incurred appreciable error relative to the reference ESP of PBE0(AC) even when atomic hexadecapoles were included in the multipole expansion. We found that the primary source of this error stemmed from insufficient higher order angular momenta blocks of the Molecular-Auxiliary basis. To address this, we developed a Molecular-Auxiliary basis (notated to as, "Pitt-fit," here and in the main document) which has two more p functions and one more d function for carbon than aug-cc-pVTZ-RI. Additionally, there is one more p, d, and f function for hydrogen than aug-cc-pVTZ-RI. As noted previously, in all our calculations the s-block of the Molecular-Auxiliary basis (aug-cc-pVTZ-RI in this case) is replaced by the ISA set2 basis. The Pitt-fit basis has two less s-functions for hydrogen than ISA set2 and one extra function for carbon. The exponents of the s-functions in our Pitt-fit basis are similar to that of ISA set2 and the exponents of the higher order angular momenta blocks are comparable to aug-cc-pVTZ-RI. However they are not exactly the same and for those interested the Pitt-fit basis set is available in the GitHub repository: https://github.com/dev-m-mulvey/quadrupole.git. Figure S1 illustrates the reduction in error in a scan of the molecular electrostatic potential above the center of mass of $C_{54}H_{18}$ when fitting the PBE0(AC) charge density with the Pitt-Fit basis.

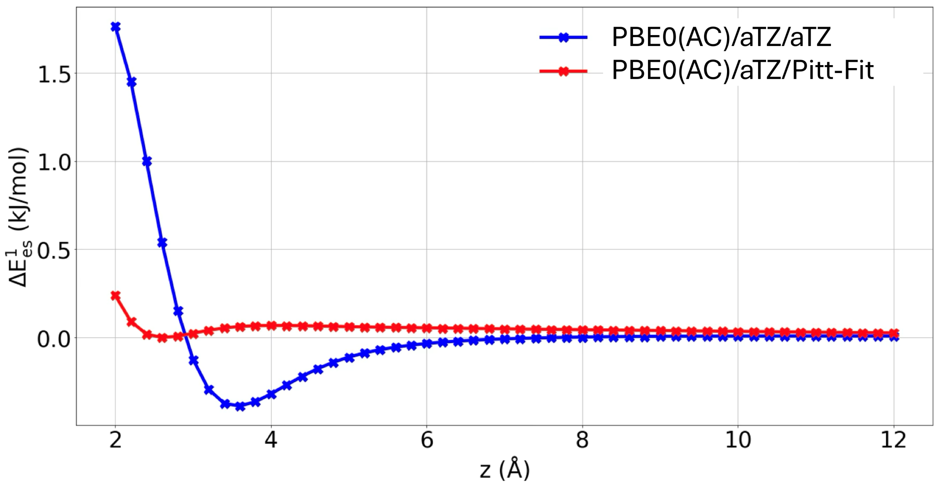

Figure S1: Error in molecular electrostatic potential at a distance z above center of $C_{54}H_{18}$ when fitting the reference PBE0(AC) charge density with the Pitt-Fit or aug-cc-pVTZ-RIfit + set2 auxiliary bases.

As described by Misquitta & Stone,[S6] the CamCASP code provides two ways of calculating the distributed multipole moments from the BS-ISA solution: one uses the basis expansions for the atoms-in-the-molecule densities, and the other grid-based approach uses only the spherically averaged, pro-atomic densities. Of these, the grid-based approach, termed 'ISA-GRID', has been shown to be more accurate,[S6] and we will use this approach here.

The atomic moments reported in the main manuscript were obtained using PBE0(AC)/aTZ/Pitt-fit/aQZ calculations. However, we wanted to report the sensitivity of the Q^{C}_{20} quadrupole component to the orbital and fitting bases, so there is data tabulated in this document for the four other combinations listed in Table S1.

2.2 Electrostatic Interaction Scans

In the main document, figure 1(a) plots the electrostatic interaction of a negative point charge with $C_{96}H_{24}$ when it is scanned along the C_6 rotational axis of the PAH. The figures plot energies for the interaction of the point charge with the PBE0(AC) charge density of $C_{96}H_{24}$ as well as the GDMA and BS-ISA multipoles parsed from that same charge density. The process for generating the data for these images went as follows:

1. Run a PBE0(AC) calculation in the basis used to generate the multipoles (aTZ orbital basis with the aTZ-JK SCF-Auxiliary basis). The steps involved in this calculation were detailed in steps 1, 2, and 3(a) of the section titled, "Methods: Calculating GDMA and BS-ISA Multipoles".

2. Calculate the electrostatic interaction with the converged all-electron density on a grid of points running along the C_6 rotational axis of the PAH (z-axis in our geometry) with a step size of $\Delta z = 0.2$ Å using the `grid_esp` feature of the one-electron properties, `oeprop`, module in Psi4 v.1.4a2.dev213.

3. Calculate the electrostatic interaction with GDMA and BS-ISA multipoles along the same grid using the program ORIENT v.5.0.08.[S32] This step was performed using a successively higher rank multipole expansion on the atoms using the `limit` command in ORIENT.

To generate the energy difference (ΔE^1_{es}) plot (figure 1(b) in the main document) we calculate the difference between the PBE0(AC) energy, $E^1_{\mathrm{es}}(\mathrm{PBE0(AC)})$, and that of the model multipolar energy, $E^1_{\mathrm{es}}(\ell_{\max})$,

$$\Delta E^1_{\mathrm{es}} = E^1_{\mathrm{es}}(\mathrm{PBE0(AC)}) - E^1_{\mathrm{es}}(\ell_{\max}) \qquad \ell_{\max} = 2, 3, 4 \tag{10}$$

In figure 1 (c) of the main document we plot charge penetration estimates at small point charge-PAH separations ($z \sim 2 - 4$ Å), as the natural log of the absolute value of ΔE^1_{es}. These results are discussed in detail in the main document, so we only add here that the short-range curves were fit with linear functions using the `curve_fit` function of the python library SciPy.[S33]

In the main text it is stated that the penetration error in the W & B electrostatic potential decays very slowly to zero for large values of z. We include figure S2 to demonstrate this. The figure plots ΔE^1_{es} for $C_{96}H_{24}$ of the BS-ISA and GDMA multipole expansions for $\ell_{max} = 2$ as well as the W & B across the full range of point charge-PAH separations $2 \leq z \leq 12$ Å.

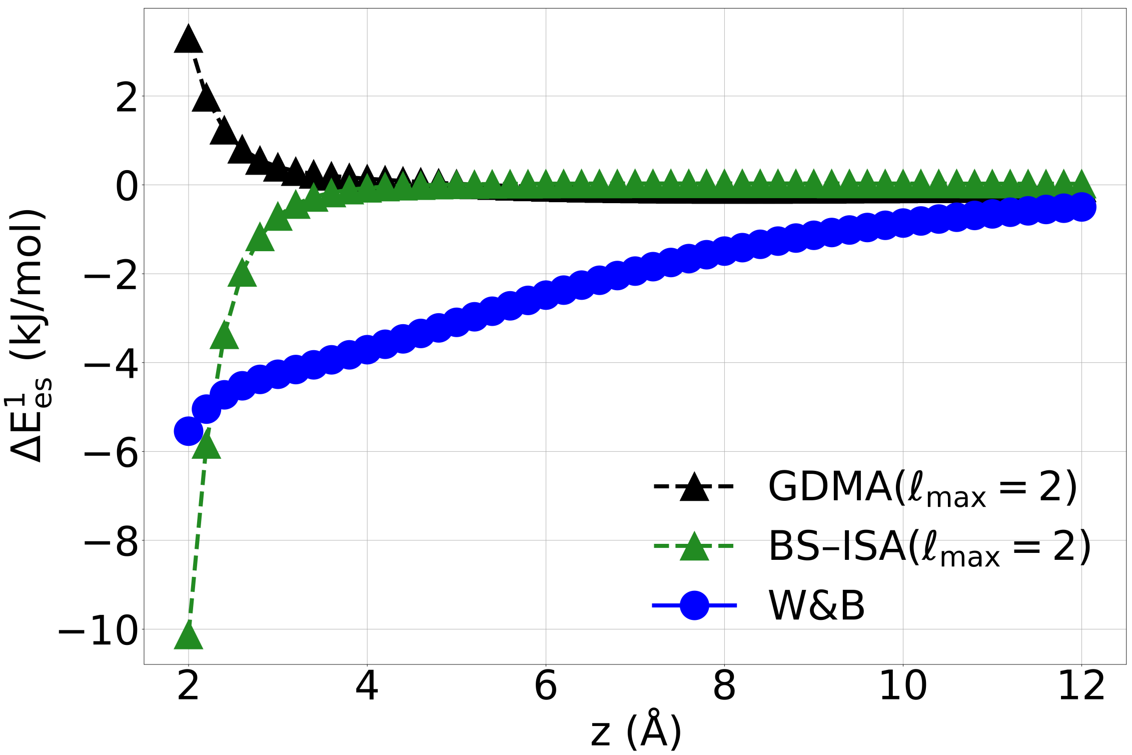

Figure S2: Penetration energy in multipolar electrostatic interaction energy ΔE^1_{es} when a negative point charge is scanned along the principle rotation axis of dicircumcoronene ($C_{96}H_{24}$). The value of ℓ_{max} in the legend indicates the highest rank atomic multipole in the expansion. For atomic multipoles derived from quantum mechanical calculations (BS-ISA and GDMA) this means atomic charges, dipoles, and quadrupoles are included. In the case of the W & B model an atomic quadrupole moment of $Q_{20} = -0.764$ a.u. was placed on every carbon atom.

As for the results where we evaluate the impact of edge dipoles on E^1_{es} between a negative point charge and increasingly large carbon nanoflakes (Figure 2 in the main document) two separate calculations were performed. For the results obtained without any edge dipoles the process went as follows:

1. Calculate the average atomic Q^C_{20} from the central six carbon atoms of $C_{24}H_{12}$, $C_{54}H_{18}$, and $C_{96}H_{24}$ given by a BS-ISA calculation on PBE0(AC)/aTZ/Pitt-fit/aQZ density. The Q^C_{20} from $C_{54}H_{18}$ and $C_{96}H_{24}$ are our best estimate of graphene's out-of-plane quadrupole, $Q_{20} = -0.006$ a.u.. For systems larger than $C_{96}H_{24}$, we presume this con-

verged Q_{20}^{C} value is representative of their out-of-plane quadrupole.

2. Generate a moments file for every hexagonal carbon nanoflake in the series $\mathrm{C}_{6\mathrm{n}^2}$ n = $2, 3, \ldots, 50$ where Q_{20}^{C} is placed on every atom and all other moments are omitted.

3. For every carbon nanoflake, read in the moments file into ORIENT v.5.0.08[S32] and calculate $\mathrm{E}_{\mathrm{es}}^{1}$ for the nanoflake and a negative point charge.

The process to generate results where both the atomic out-of-plane quadrupole Q_{20}^{C} and edge dipoles (Q_{1m}^{CH}) are present went as follows:

1. Construct Q_{1m}^{CH} for C_{24}, C_{54}, and C_{96} such that the combination of Q_{20}^{C} on every carbon atom and Q_{1m}^{CH} on only the edge carbon atoms of the nanoflakes reproduce the molecular out-of-plane quadrupole of the PAHs $C_{24}H_{12}$, $C_{54}H_{18}$, and $C_{96}H_{24}$ when the $\ell_{\mathrm{max}} = 2$ expansion (atomic charges, dipoles, and quadrupoles) of BS-ISA is used.

2. Plot the edge dipole magnitude of C_{24}, C_{54}, and C_{96} as a function of 1/r where r is the average distance of an edge carbon from the center of mass of the PAHs (determined using a nearest-neighbors algorithm).

3. Fit the function $Q_{1m}^{\mathrm{CH}}(1/r) = a*(1/r)+b$ to the data using the Python library NumPy's[S34] `polyfit` function and extrapolate to obtain edge dipole magnitudes for the hexagonal carbon nanoflakes in the series $\mathrm{C}_{6\mathrm{n}^2}$ n = $2, 3, \ldots, 50$. Figure S3 plots the results of the extrapolation procedure and the edge dipoles we constructed.

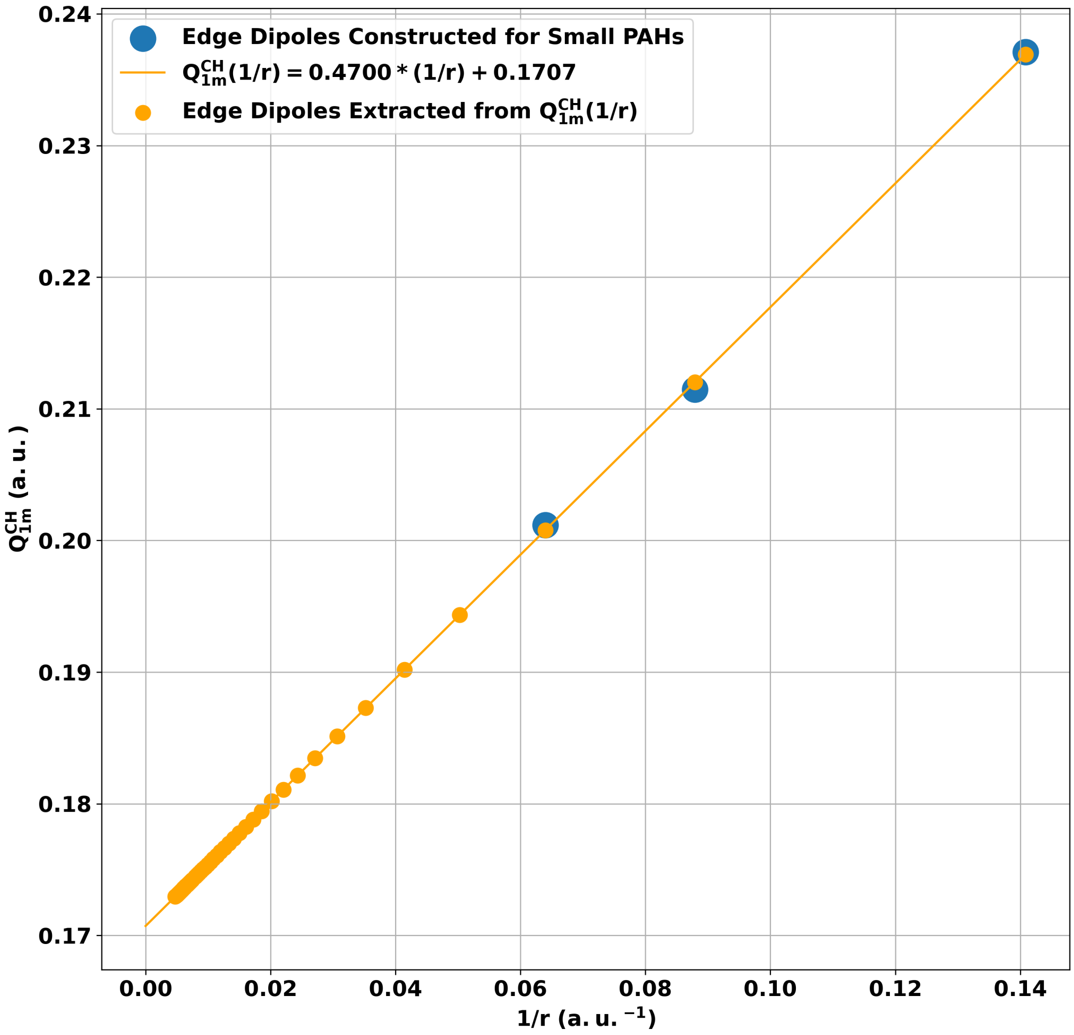

Figure S3: Plot of model edge dipole magnitudes (Q^{CH}_{1m}) as a function of $1/r$ where r is the average radius of a nanoflake. The blue points are the edge dipoles we constructed to reproduce the molecular out-of-plane quadrupole component of PAHs $C_{24}H_{12}$, $C_{54}H_{18}$, and $C_{96}H_{24}$. The function $Q^{\mathrm{CH}}_{1m}(1/r)$ was fit to the blue points and the orange points are the model edge dipoles extracted from the function.

4. Generate a moments file for the hexagonal carbon nanoflakes C_{6n^2} n = $2, 3, \ldots, 50$ where Q^{C}_{20} is placed on every atom and the edge dipoles determined in the previous step are applied to the edge carbon atoms with the correct orientation. In this context, "correct

orientation," refers to the fact that the edge bond dipoles from BS-ISA point along the C-H bond towards the position of where a hydrogen atom would be in a PAH of the same size.

5. For each carbon nanoflake considered, read in the moments file into ORIENT v.5.0.08[S32] and calculate E^1_{es} for the nanoflake and a negative point charge.

The scripts used to calculate the average edge dipole, to calculate the average distance of an edge carbon in a PAH and nanoflake, and plot the ESP scan can all be found as Jupyter Notebooks in the GitHub repository.

2.3 Calculating the Primitive Cartesian zz component of Graphene's Carbon in Periodic Boundary Conditions

We used the Castep[S35] code to perform periodic boundary condition plane-wave DFT calculations on graphene. A two-atom unit cell was employed, with a large extent of vacuum in the direction perpendicular to the sheet so as to minimise unwanted interactions between the sheet and its periodic images. The extent of the cell perpendicular to the sheet was 14Å , and we tested convergence for this parameter by reducing it to 12Å and increasing to 16Å , which had negligible effect on the result. A 7x7x2 k-space sampling grid was used for most calculations, with convergence checked by increasing this to 9x9x2 and 11x11x2. A lattice parameter of just over 4.644 a.u. was used, and relaxation was not permitted. This approximation was justified by permitting relaxation for one calculation, which resulted in the lattice parameter increasing to 4.658 a.u. The component of the quadrupole moment of interest changed from -3.922 a.u. to -3.906 a.u. per (carbon) atom.

Castep's automatically-generated pseudopotentials were employed, and we used the LDA,[S36] PW91,[S37] PBE,[S38,S39] and PBE0[S10,S11] functionals. Both norm-conserving[S40] and ultrasoft[S41] pseudopotentials were used. All produced very similar results for the $\langle zz^a \rangle$ component of the quadrupole moment, ranging from -3.922 a.u. to -3.9510 a.u. per atom.

3 Additional Results

Here we aggregate additional results of interest which were not shown in the main text to maintain focus and brevity. This includes:

- The average charge, dipole magnitude, quadrupole magnitude, and Q_{20} component on all symmetry equivalent sets of atoms in PAHs $C_{6n^2}H_{6n}$ $(n : n = 1 - 4)$.
- A plot demonstrating the oscillation of atomic charges on symmetry equivalent sets of atoms in $C_{96}H_{24}$.
- The full derivation for a point charge interacting with a 2D sheet of quadrupolar and edge ring of dipolar density,

$$V(d) = \pi(\sigma_{20} - 2\sigma_{10})\left[\frac{r^2}{(d^2 + r^2)^{3/2}}\right]$$

which appears in the main manuscript.

3.1 Tables of Average Multipole Moments on Symmetry Equivalent Atoms

Table S2: Average atomic charge (q) and atomic dipole magnitude ($\|\mu\|$) of symmetry equivalent atom sets (C_1, C_2, ..., H_{dcc2}) in PAHs $C_{6n^2}H_{6n}$ n=1–4 calculated using using the BS-ISA method with PBE0(AC)/aTZ/Pitt-fit/aQZ density.

Avg.Moment[1]	q				$\|\mu\|$			
Atom	C_6H_6	$C_{24}H_{12}$	$C_{54}H_{18}$	$C_{96}H_{24}$	C_6H_6	$C_{24}H_{12}$	$C_{54}H_{18}$	$C_{96}H_{24}$
C_1	-0.126	-0.001	0.001	-0.001	0.056	0.005	0.003	0.001
C_2		0.125	-0.007	0.000		0.000	0.002	0.002
C_3		-0.198	-0.006	0.001		0.027	0.005	0.003
C_4			0.151	-0.006			0.010	0.001
C_5			-0.302	-0.003			0.007	0.002
C_6			-0.205	-0.006			0.028	0.002
C_7				0.149				0.004
C_8				0.148				0.017
C_9				-0.292				0.015
C_{10}				-0.204				0.031
H_b[2]	0.126				0.048			
H_c		0.136				0.045		
H_{cc1}			0.147				0.041	
H_{cc2}			0.140				0.044	
H_{dcc1}				0.146				0.041
H_{dcc2}				0.140				0.044

[1] All multipoles are spherical, traceless, and in atomic units.
[2] Symmetry equivalent sets of hydrogen atoms on PAHs are designated by their subscripts: b → benzene, c → coronene, cc# → circumcoronene, and dcc# → dicircumcoronene.

Table S3: Average atomic charge (q) and atomic dipole magnitude ($\|\mu\|$) of symmetry equivalent atom sets (C_1, C_2, ..., H_{dcc2}) in PAHs $C_{6n^2}H_{6n}$ n=1–4 calculated using the GDMA method with PBE0(AC)/aTZ density.

Avg.Moment	q				$\|\mu\|$			
Atom	C_6H_6	$C_{24}H_{12}$	$C_{54}H_{18}$	$C_{96}H_{24}$	C_6H_6	$C_{24}H_{12}$	$C_{54}H_{18}$	$C_{96}H_{24}$
C_1	-0.093	-0.008	-0.001	-0.001	0.120	0.017	0.002	0.001
C_2		-0.045	-0.002	-0.001		0.111	0.011	0.000
C_3		-0.076	-0.007	-0.001		0.166	0.012	0.003
C_4			-0.043	-0.002			0.119	0.011
C_5			-0.065	-0.007			0.163	0.007
C_6			-0.075	-0.008			0.172	0.010
C_7				-0.041				0.123
C_8				-0.043				0.125
C_9				-0.064				0.164
C_{10}				-0.074				0.175
H_b [1]	0.093				0.130			
H_c		0.103				0.135		
H_{cc1}			0.110				0.139	
H_{cc2}			0.105				0.134	
H_{dcc1}				0.111				0.138
H_{dcc2}				0.105				0.134

[1] See Table S2 for a full description of all the conventions used for labeling symmetry equivalent hydrogen atoms.

Table S4: Average atomic quadrupole Q_{20} component and magnitude of quadrupole $\|Q_{2m}\|$ on symmetry equivalent atom sets calculated using the BS-ISA method with PBE0(AC)/aTZ/Pitt-fit/aQZ density.

Avg.Moment	Q_{20}				$\|Q_{2m}\|$[2]			
Atom	C_6H_6	$C_{24}H_{12}$	$C_{54}H_{18}$	$C_{96}H_{24}$	C_6H_6	$C_{24}H_{12}$	$C_{54}H_{18}$	$C_{96}H_{24}$
C_1	0.007	-0.019	-0.006	-0.006	0.072	0.021	0.006	0.006
C_2		0.018	-0.007	-0.007		0.035	0.007	0.008
C_3		0.021	-0.017	-0.006		0.031	0.018	0.006
C_4			0.012	-0.008			0.022	0.008
C_5			0.029	-0.011			0.029	0.012
C_6			0.022	-0.017			0.032	0.019
C_7				0.011				0.012
C_8				0.013				0.023
C_9				0.026				0.026
C_{10}				0.026				0.038
H_b[1]	-0.035				0.035			
H_c		-0.031				0.031		
H_{cc1}			-0.021				0.022	
H_{cc2}			-0.030				0.030	
H_{dcc1}				-0.023				0.023
H_{dcc2}				-0.030				0.030

[1] See Table S2 for a full description of all the conventions used for labeling symmetry equivalent hydrogen atoms.

[2] In $\|Q_{2m}\|$, the $_m$ denotes that all components m = 0, 1c, 1s, 2c, 2s of the spherical quadrupole tensor are included in the magnitude. All multipoles are spherical, traceless, and in atomic units.

Table S5: Average atomic quadrupole Q_{20} component and magnitude of quadrupole $\|Q_{2m}\|$ on symmetry equivalent atom sets calculated using GDMA multipoles were parsed from PBE0(AC)/aTZ density.

Avg.Moment	Q_{20}				$\|Q_{2m}\|^2$			
Atom	C_6H_6	$C_{24}H_{12}$	$C_{54}H_{18}$	$C_{96}H_{24}$	C_6H_6	$C_{24}H_{12}$	$C_{54}H_{18}$	$C_{96}H_{24}$
C_1	-1.137	-1.158	-1.168	-1.169	1.141	1.158	1.168	1.169
C_2		-1.132	-1.163	-1.169		1.136	1.163	1.169
C_3		-1.155	-1.165	-1.168		1.155	1.165	1.168
C_4			-1.136	-1.164			1.142	1.164
C_5			-1.159	-1.170			1.164	1.170
C_6			-1.157	-1.167			1.157	1.167
C_7				-1.139				1.145
C_8				-1.138				1.143
C_9				-1.161				1.166
C_{10}				-1.157				1.157
H_b[1]	-0.151				0.185			
H_c		-0.148				0.167		
H_{cc1}			-0.145				0.154	
H_{cc2}			-0.148				0.166	
H_{dcc1}				-0.145				0.153
H_{dcc2}				-0.148				0.165

[1] See Table S2 for a full description of all the conventions used for labeling symmetry equivalent hydrogen atoms.
[2] See Table S4 for a description of the meaning of m in $\|Q_{2m}\|$.

Table S6: Average atomic quadrupole Q_{20} component and magnitude of quadrupole $\|Q_{2m}\|$ on symmetry equivalent atom sets (C_1, C_2, ..., H_c) in PAHs $C_{6n^2}H_{6n}$ n = 1 – 2 calculated using BS-ISA and GDMA partitioned from PBE0(AC) density[1].

	BS-ISA aDZ/aDZ/aDZ				GDMA aDZ			
Avg.Moment	Q_{20}		$\|Q_{2m}\|$[2]		Q_{20}		$\|_{2m}\|$	
Atom	C_6H_6	$C_{24}H_{12}$	C_6H_6	$C_{24}H_{12}$	C_6H_6	$C_{24}H_{12}$	C_6H_6	$C_{24}H_{12}$
C_1	0.001	-0.086	0.077	0.086	-1.169	-1.195	1.173	1.195
C_2		-0.028		0.040		-1.167		1.171
C_3		-0.030		0.049		-1.184		1.184
H_b[3]	-0.030		0.034		-0.141		0.182	
H_c		-0.022		0.029		-0.142		0.164
	BS-ISA aDZ/aDZ/aTZ				GDMA aDZ			
Avg.Moment	Q_{20}		$\|Q_{2m}\|$		Q_{20}		$\|Q_{2m}\|$	
Atom	C_6H_6	$C_{24}H_{12}$	C_6H_6	$C_{24}H_{12}$	C_6H_6	$C_{24}H_{12}$	C_6H_6	$C_{24}H_{12}$
C_1	0.001	-0.086	0.077	0.086	-1.169	-1.195	1.173	1.195
C_2		-0.028		0.040		-1.167		1.171
C_3		-0.030		0.049		-1.184		1.184
H_b	-0.030		0.034		-0.141		0.182	
H_c		-0.022		0.029		-0.142		0.164
	BS-ISA aTZ/aTZ/aTZ				GDMA aTZ			
Avg.Moment	Q_{20}		$\|Q_{2m}\|$		Q_{20}		$\|Q_{2m}\|$	
Atom	C_6H_6	$C_{24}H_{12}$	C_6H_6	$C_{24}H_{12}$	C_6H_6	$C_{24}H_{12}$	C_6H_6	$C_{24}H_{12}$
C_1	0.042	-0.023	0.076	0.024	-1.137	-1.158	1.141	1.158
C_2		0.009		0.028		-1.132		1.136
C_3		0.018		0.028		-1.155		1.155
H_b	-0.034		0.035		-0.151		0.185	
H_c		-0.029		0.029		-0.148		0.167
	BS-ISA aTZ/aTZ/aQZ				GDMA aTZ			
Avg.Moment	Q_{20}		$\|Q_{2m}\|$		Q_{20}		$\|Q_{2m}\|$	
Atom	C_6H_6	$C_{24}H_{12}$	C_6H_6	$C_{24}H_{12}$	C_6H_6	$C_{24}H_{12}$	C_6H_6	$C_{24}H_{12}$
C_1	0.042	-0.023	0.076	0.024	-1.137	-1.158	1.141	1.158
C_2		0.009		0.028		-1.132		1.136
C_3		0.018		0.028		-1.155		1.155
H_b	-0.034		0.035		-0.151		0.185	
H_c		-0.029		0.029		-0.148		0.167
	BS-ISA aTZ/Pitt-fit/aQZ				GDMA aTZ			
Avg.Moment	Q_{20}		$\|Q_{2m}\|$		Q_{20}		$\|Q_{2m}\|$	
Atom	C_6H_6	$C_{24}H_{12}$	C_6H_6	$C_{24}H_{12}$	C_6H_6	$C_{24}H_{12}$	C_6H_6	$C_{24}H_{12}$
C_1	0.007	-0.019	0.072	0.021	-1.137	-1.158	1.141	1.158
C_2		0.018		0.035		-1.132		1.136
C_3		0.021		0.031		-1.155		1.155
H_b	-0.035		0.035		-0.151		0.185	
H_c		-0.031		0.031		-0.148		0.167

[1] The PBE0(AC) density was constructed from the basis used is indicated in the table.
[2] See Table S4 for a description of the meaning of m in $\|Q_{2m}\|$.
[3] See Table S2 for a full description of all the conventions used for labeling symmetry equivalent hydrogen atoms.

3.2 Charge Oscillations

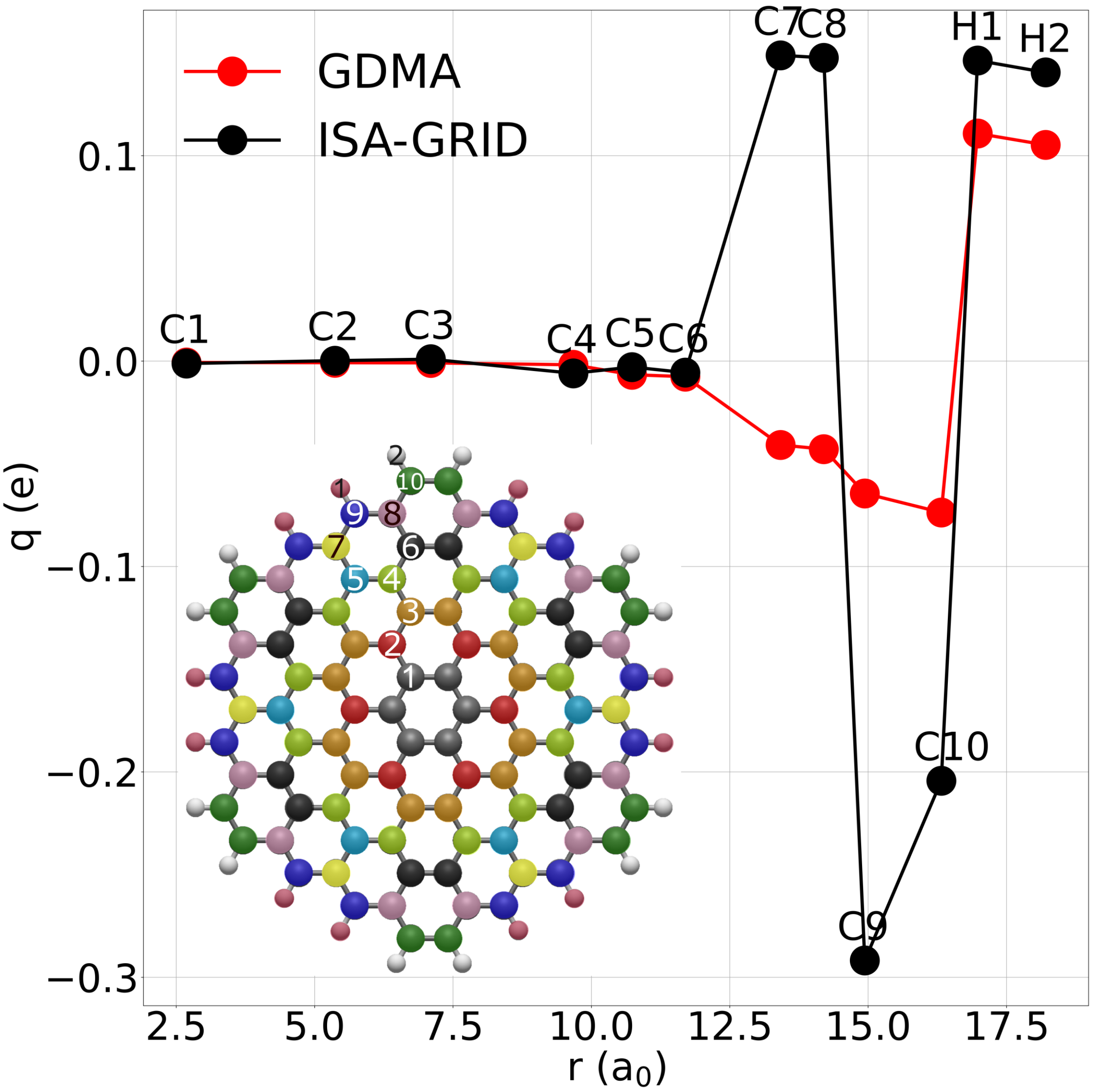

Figure S4: Plot of BS-ISA and GDMA atomic charges as a function of r, where r is the distance of a set of symmetry equivalent atoms (C_1, C_2, ..., H_2) from the COM in PAH $C_{96}H_{24}$. Embedded is an image showing the symmetry equivalent sets of atoms coded by color and numerical label that corresponds to those used in the scatter plot.

3.3 Derivation of Electrostatic Interaction of a Point Charge with a Finite Disk of Quadrupolar and Edge Dipolar Density

The full derivation of equation 1 in the main manuscript requires defining the energy of a point charge interacting with a single point Q_{20} quadrupole component as well as the energy of a point charge interacting with a single point dipole. Once, the energy of these quantities are defined, we can consider how to extend this to the interaction with a continuum of quadrupolar density and ring of dipolar density.

To define the interaction energy between a point charge and point Q_{20} or point dipole, one needs to define the coordinate system. We will do this first for the global axis system, which is shown for both the point multipoles being considered in figure S5.

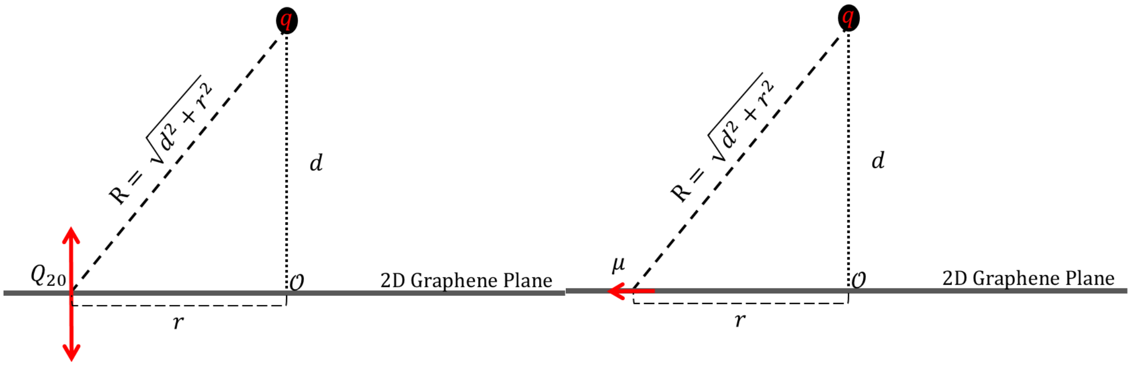

Figure S5: Schematics illustrating the arrangement of a point charge (q) interacting with: (Left) A Q_{20} quadrupole component on one of the carbon atoms in a 2D graphene plane. (Right) The μ dipole component on one of the edge CH pair of a 2D PAH.

We define some point charge to lie at a distance d above the origin ($\mathcal{O}$) of a 2D plane representing a graphene sheet, which contains either the point quadrupole component (Q_{20}) or point dipole μ some distance r away within the graphene plane. By simple geometry this arrangement can be shown to form a right triangle with a hypotenuse of,

$$R = \sqrt{d^2 + r^2} \tag{11}$$

which represents the distance between the point multipole and point charge, R.

To evaluate the interaction between these point multipoles we will be using Stone's tensor mathematics formulation,[S4] due to the compactness and simplicity of it's notation. If we want to evaluate the electrostatic interaction energy ($E^{ab}_{\mathrm{t,u}}$) between two charge densities a and b with multipoles t and u respectively, then the expression can be written as,

$$E^{ab}_{\mathrm{t,u}} = Q^a_t T_{t,u} Q^b_u \tag{12}$$

where Q^a_t and Q^b_u are charge densities a and b with multipoles t and u respectively and $T_{t,u}$ is the electrostatic interaction tensor for all multipoles describing charge densities a and b. Note that t and u are indices representative of **all** multipole moments centered on the charge densities. These indices represent spherical harmonic components of the traceless multipole tensors ($t = 00, 10, 11c, 11s, 20, \ldots, \ell k$) where ℓ represents rank of multipole (0,1,2,... → charge, dipole, quadrupole,...) and k the component of said multipole rank (1c,1s,0 → x,y,z).

Explaining all the details of how this formulation/notation of electrostatics works lies outside the scope of this document and those interested should refer to Stone's book.[S4] That being said, the simplicity of this system and its inherent symmetries allow us to make simplifications tying the spherical harmonic formulation directly back to Cartesian coordinates.

3.3.1 Charge Interacting with Plane of Quadrupolar Density

First we will address evaluating the electrostatic energy of the point charge and Q_{20} interaction. From Stone's book the interaction tensor for a charge and the z-component of quadrupole (Q_{20}) is,

$$T_{20,00} = \frac{1}{2R^4}\left(3r_{zz}^2 - 1\right) \tag{13}$$

where R is the distance between the charge and quadrupole and r_{zz} is the extent of the zz-component quadrupole when projected from the local axis frame onto that of the global axis frame we defined in figure S5. The local axis frame refers to a coordinate system that

defines the alignment of the x, y, and z components of the multipoles with respect to the atomic center, which are independent of the global coordinate system.

Since a point charge is spherical and can described through scalar quantities, we can just say that the local coordinate system aligns with whatever serves our purpose. That leaves defining a local coordinate system for Q_{20}. For that purpose we define a unit vector $\hat{n}^a$ for the quadrupole that aligns with its zz-component ($\hat{e}_z^a$), which is its only non-zero component.

The left hand image of figure S6 illustrates the relationship between this unit vector $\hat{n}^a$ with origin at the center of Q_{20} to the interaction axis with magnitude R. If we define the vector connecting the center of the quadrupole to the point charge, $\hat{R}$, to correspond to our global z-axis ($\hat{z}$), then we only need to know the angle between the unit vector $\hat{n}^a$ and $\hat{R}$, which is ϑ_a. In the right hand image of figure S6 illustrates where ϑ_a lies within the global axis system picture initially shown in figure S5.

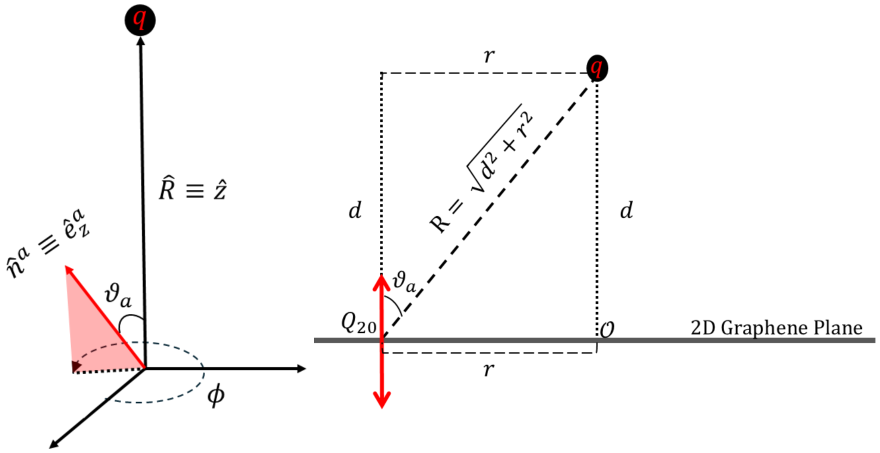

Figure S6: Schematic illustrating the definition of local axes of point quadrupole moment (red arrow) relative to global axes frame.

With this definition of local and global coordinate systems r_{zz} within equation 13 becomes,

$$T_{20,00} = \frac{1}{2R^3}\left(3\left(\hat{n}^a \cdot \hat{R}\right)^2 - 1\right) \tag{14}$$

where $\hat{n}^a \cdot \hat{R}$ simply represents the projection of unit vector corresponding to the zz-direction of the point quadrupole onto the vector $\hat{R}$. Since, $\hat{R}$ is defined to be $\hat{z}$ we have the relation-

ship:

$$\hat{n}^a \cdot \hat{R} = \hat{n}^a \cdot \hat{z} = \cos(\vartheta_a) \tag{15}$$

With the directional dependence handled all we need to do is insert the magnitude of our quadrupole Q_{20} and charge (q) to obtain the electrostatic interaction energy.

$$E_{20,00} = \frac{Q_{20}q}{2R^3}\left(3\cos^2(\vartheta_a) - 1\right) \tag{16}$$

Which should look familiar to some. It may seem pedantic to the reader that we go through the trouble of deriving this expression, but setting the coordinate system up is crucial for what is to follow.

Now, we will transform equation 16 to represent the interaction of point charge with a slice of quadrupolar surface density. First, we must define the magnitude Q_{20} as a portion of graphene's quadrupolar surface density. We define the relationship between magnitude of point quadrupole Q_{20} and magnitude of quadrupolar surface density (σ_{20}),

$$Q_{20} = \sigma_{20}\Delta A \tag{17}$$

Where ΔA is the area that a carbon atom occupies in graphene. We are essentially matching the magnitude of point quadrupole Q_{20} to that of a slice of graphene's quadrupolar density with area ΔA. If we assume the graphene sheet to be a 2D disk with radius r, which is appropriate given the hexagonal shape of PAHs and graphene nanoflakes studied in this work, we arrive at the following expression for ΔA

$$\Delta A = 2\pi r\Delta r \tag{18}$$

Inserting this into our electrostatic energy expression (equation 16) we arrive at,

$$\Delta E_{20,00} = \frac{(\sigma_{20}2\pi r\Delta r)q}{2R^3}\left(3\cos^2\vartheta_a - 1\right) \tag{19}$$

where all terms have been defined previously. To evaluate the interaction energy of the point charge will *all* slices of quadrupolar density within the disk of the graphene nanoflake we must integrate the expression with respect to radius r like so,

$$E_{20,00} = \int dE_{20,00} = \int_0^{r_0} \frac{(\sigma_{20} 2\pi r) q}{2R^3} \left(3\cos^2\vartheta_a - 1\right) dr \tag{20}$$

where we take 0 to be the center of the graphene disk and r_0 to be the full radius of the disk. To integrate this expression we need to make the dependence of all terms on r explicit. We refer back to the right image in figure S6 to define $\cos(\vartheta_a)$ in terms of r,

$$\cos(\vartheta_a) = \frac{d}{R} = \frac{d}{\sqrt{d^2 + r^2}} \tag{21}$$

where this definition comes completely from elementary trigonometry relationships. Substituting this into the energy integral expression, simplifying, and integrating with respect to r yields the following.

$$E_{20,00} = \sigma_{20}\pi q \int_0^{r_0} \frac{r}{(d^2 + r^2)^{3/2}} \left(\frac{3d^2}{d^2 + r^2} - 1\right) dr = \sigma_{20}\pi q \left[\frac{r_0^2}{\left(d^2 + r_0^2\right)^{3/2}}\right] \tag{22}$$

Taking the limit of this expression as $r_0 \to \infty$ gives us,

$$\lim_{r_0 \to \infty} \sigma_{20}\pi q \left[\frac{r_0^2}{\left(d^2 + r_0^2\right)^{3/2}}\right] = \sigma_{20}\pi q \left[\frac{1}{r_0}\right] \tag{23}$$

which demonstrates the slow convergence of the interaction energy to zero as the radius of the finite graphene disk approaches infinity, which is expected and has been demonstrated through other methods.

3.3.2 Charge Interacting with Edge Ring of Dipolar Density

Now we will derive the expression for a ring of dipolar density at the edge of the finite graphene nanoflake. As before, we need to determine the interaction of a charge with a

point dipole given the coordinate system shown in the right image of S5. From Stone's book the interaction tensor for a charge and the z-component of dipole (μ_{10}) is,

$$T_{00,10} = \frac{r_z}{R^2} \tag{24}$$

where r_z is the extent of the z-component dipole when projected from the local axis frame onto that of the global axis frame we defined in figure S5.

We define a unit vector $\hat{n}^b$ for the dipole that aligns with its z-component ($\hat{e}_z^b$), which is its only non-zero component. The relationship between the local axes of the dipole and the global axes are similar to that of the problem involving the quadrupole except for a few key aspects. The left hand image of figure S7 illustrates the relationship between this unit vector $\hat{n}^b$ with its origin at the center of μ_{10} and the global axes.

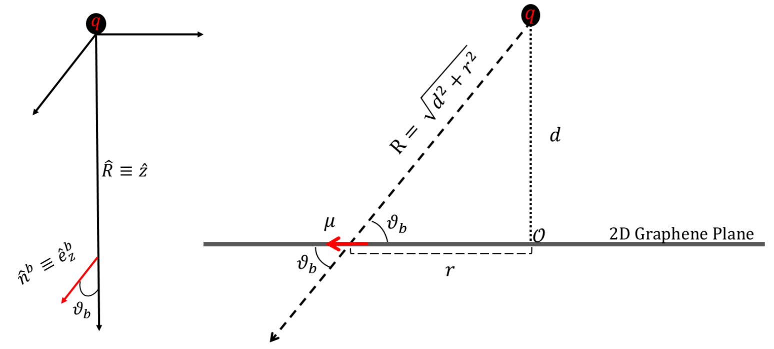

Figure S7: Schematic illustrating the definition of local axes of point dipole moment (red arrow) relative to global axes frame.

In this instance it, we take the origin of the global coordinate system to be at the point charge and the interaction axis with magnitude R to coincide with $\hat{z}$ in the global frame. Again, by this definition we only need to know the angle between the unit vector $\hat{n}^b$ and $\hat{R}$, which is ϑ_b. This definition of local and global coordinate systems yields the following relationship between these quantities,

$$\hat{n}^b \cdot \hat{R} = -\hat{n}^b \cdot \hat{z} = -\cos(\vartheta_b) \tag{25}$$

where we have the negative cosine of ϑ_b because we chose the origin of the global frame to lie at the point charge with $\hat{R}$ pointing in the direction of the dipole. This leads to the following expression for the interaction energy,

$$E_{00,10} = \frac{-q\mu_{10}\cos(\vartheta_b)}{R^2} \tag{26}$$

which should look familiar once again as it is just the energy of a point charge interacting with a point dipole. We can write out this functions dependence on r more explicitly by recognizing that,

$$\cos(\vartheta_b) = \frac{r}{R} = \frac{r}{\sqrt{d^2 + r^2}} \tag{27}$$

which means equation 26 can be written as such.

$$E_{00,10} = \frac{-q\mu_{10}r}{(d^2 + r^2)^{3/2}} \tag{28}$$

Now, we will transform equation 26 to represent the interaction of point charge with a ring of dipolar density at the edge of large polycyclic aromatic hydrocarbon. First, we must define the magnitude μ_{10} as a portion of PAH's edge dipolar density. We define the relationship between magnitude of point dipole μ_{10} and magnitude of edge dipolar density σ_{10} as,

$$\mu_{10} = \sigma_{10}\Delta l \tag{29}$$

where Δl is the arc-length associated with a terminating CCHCH group and μ_{10} is the dipole associated with this group. This terminating CCHCH group functions as a unit cell for the structure of edge of the PAH. To better understand what this formula represents, refer to figure S8. As shown in figure S8, is a slice of the ring of edge dipolar density with arc length Δl. This can be directly equated to the product of r_0 and $\Delta\varphi$, which are the radius and

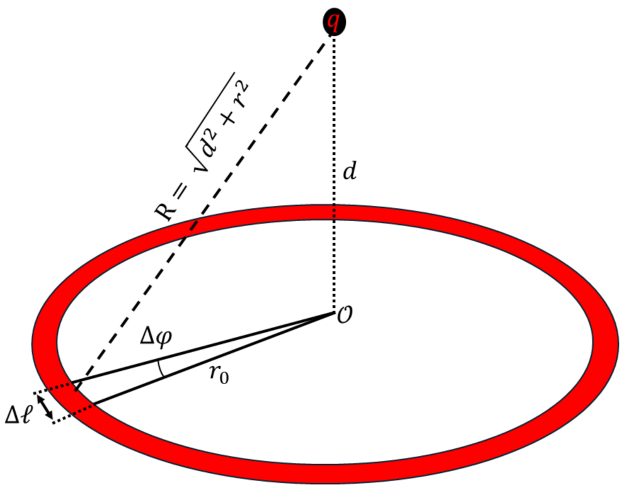

Figure S8: Integration strategy for the charge..edge-dipole interaction.

central angle associated with the arc length.

$$\sigma_{10}\Delta l = \sigma_{10} r_0 \Delta\varphi \tag{30}$$

We can substitute this directly into equation 28.

$$\Delta E_{00,10} = \frac{-\sigma_{10} r_0^2 q}{\left(d^2 + r_0^2\right)^{3/2}} \Delta\varphi \tag{31}$$

To evaluate the interaction energy of the point charge with the entire ring of dipolar density at the edge of the PAH, we must integrate the expression with respect to angle φ like so,

$$E_{00,10} = \int dE_{00,10} = \int_0^{2\pi} \frac{-\sigma_{10} r_0^2 q}{\left(d^2 + r_0^2\right)^{3/2}} d\varphi = -2\pi\sigma_{10} q \left[\frac{r_0^2}{\left(d^2 + r_0^2\right)^{3/2}}\right] \tag{32}$$

Which has the same functional form as equation 22 save the negative constant outside the brackets. This means the limiting behavior with respect to r_0 is the same.

$$\lim_{r_0 \to \infty} -2\pi\sigma_{10} q \left[\frac{r_0^2}{\left(d^2 + r_0^2\right)^{3/2}}\right] = -2\pi\sigma_{10} q \left[\frac{1}{r_0}\right] \tag{33}$$

3.3.3 Charge Interacting with Plane of Quadrupolar Density Terminated by a Ring of Dipolar Density

Lastly, we sum equations 22 and 32 to show the electrostatic energy of a point charge interacting with a disk of quadrupolar density terminated by a ring of dipolar density, which is the equation shown in the main manuscript.

$$E_T = \pi q\left(\sigma_{20} - 2\sigma_{10}\right)\left[\frac{r_0^2}{\left(d^2 + r_0^2\right)^{3/2}}\right] \tag{34}$$

Which has the expected limiting behavior with respect to r_0.

$$\lim_{r_0 \to \infty} E_T = \pi q\left(\sigma_{20} - 2\sigma_{10}\right)\left[\frac{1}{r_0}\right] \tag{35}$$

References

(S1) Granger, B. E.; Perez, F. Jupyter: Thinking and Storytelling with Code and Data. *Comput. Sci. Eng.* **2021**, *23*, 7–14.

(S2) Buckingham, A. D. Molecular quadrupole moments. *Q. Rev., Chem. Soc.* **1959**, *13*, 183–214.

(S3) Buckingham, A. D. Permanent and Induced Molecular Moments and Long-Range Intermolecular Forces. In *Adv. Chem. Phys.*; Hirschfelder, J., Ed.; John Wiley & Sons, Ltd, 1967; Chapter 2, pp 107–142.

(S4) Stone, A. *The Theory of Intermolecular Forces*, 2nd ed.; 2013.

(S5) Misquitta, A. J.; Stone, A. J.; Fazeli, F. Distributed multipoles from a robust basis-

space implementation of the iterated stockholder atoms procedure. *J. Chem. Theory Comput.* **2014**, *10*, 5405–5418.

(S6) Misquitta, A. J.; Stone, A. J. ISA-Pol: distributed polarizabilities and dispersion models from a basis-space implementation of the iterated stockholder atoms procedure. *Theor. Chem. Acc.* **2018**, *137*, 1–20.

(S7) Stone, A. J. Distributed multipole analysis, or how to describe a molecular charge distribution. *Chem. Phys. Lett.* **1981**, *83*, 233–239.

(S8) Stone, A. J.; Alderton, M. Distributed multipole analysis. *Molecular Physics* **1985**, *56*, 1047–1064.

(S9) Stone, A. J. Distributed Multipole Analysis: Stability for Large Basis Sets. *J. Chem. Theory Comput.* **2005**, *1*, 1128–1132.

(S10) Adamo, C.; Barone, V. Toward reliable density functional methods without adjustable parameters: The PBE0 model. *J. Chem. Phys.* **1999**, *110*, 6158.

(S11) Perdew, J. P.; Ernzerhof, M.; Burke, K. Rationale for mixing exact exchange with density functional approximations. *J. Chem. Phys.* **1996**, *105*, 9982–9985.

(S12) Stanton, J. F.; Gauss, J. Analytic energy derivatives for ionized states described by the equation-of-motion coupled cluster method. *J. Chem. Phys.* **1994**, *101*, 8938.

(S13) Dutta, A. K.; Saitow, M.; Riplinger, C.; Neese, F.; Izsák, R. A near-linear scaling equation of motion coupled cluster method for ionized states. *J. Chem. Phys.* **2018**, *148*, 244101.

(S14) Neese, F.; Wennmohs, F.; Becker, U.; Riplinger, C. The ORCA quantum chemistry program package. *J. Chem. Phys.* **2020**, *152*, 224108.

(S15) Hartree, D. R. The Wave Mechanics of an Atom with a Non-Coulomb Central Field. Part I. Theory and Methods. *Math. Proc. Cambridge Philos. Soc.* **1928**, *24*, 89–110.

(S16) Fock, V. Näherungsmethode zur Lösung des quantenmechanischen Mehrkörperproblems. *Zeitschrift für Physik* **1930**, *61*, 126–148.

(S17) Roothaan, C. C. J. New Developments in Molecular Orbital Theory. *Rev. Mod. Phys* **1951**, *23*, 69.

(S18) Dunning, T. H. Gaussian basis sets for use in correlated molecular calculations. I. The atoms boron through neon and hydrogen. *J. Chem. Phys.* **1989**, *90*, 1007.

(S19) Neese, F.; Wennmohs, F.; Hansen, A.; Becker, U. Efficient, approximate and parallel Hartree–Fock and hybrid DFT calculations. A 'chain-of-spheres' algorithm for the Hartree–Fock exchange. *Chem. Phys.* **2009**, *356*, 98–109.

(S20) Izsák, R.; Neese, F. An overlap fitted chain of spheres exchange method. *J. Chem. Phys.* **2011**, *135*, 144105.

(S21) Weigend, F. Accurate Coulomb-fitting basis sets for H to Rn. *Phys. Chem. Chem. Phys.* **2006**, *8*, 1057–1065.

(S22) Izsák, R.; Hansen, A.; Neese, F. The resolution of identity and chain of spheres approximations for the LPNO-CCSD singles Fock term. *Molecular Physics* **2012**, *110*, 2413–2417.

(S23) Weigend, F.; Köhn, A.; Hättig, C. Efficient use of the correlation consistent basis sets in resolution of the identity MP2 calculations. *J. Chem. Phys.* **2002**, *116*, 3175.

(S24) Parrish, R. M.; Burns, L. A.; Smith, D. G.; Simmonett, A. C.; DePrince, A. E.; Hohenstein, E. G.; Bozkaya, U.; Sokolov, A. Y.; Di Remigio, R.; Richard, R. M.; Gonthier, J. F.; James, A. M.; McAlexander, H. R.; Kumar, A.; Saitow, M.; Wang, X.; Pritchard, B. P.; Verma, P.; Schaefer, H. F.; Patkowski, K.; King, R. A.; Valeev, E. F.; Evangelista, F. A.; Turney, J. M.; Crawford, T. D.; Sherrill, C. D. Psi4 1.1: An Open-

Source Electronic Structure Program Emphasizing Automation, Advanced Libraries, and Interoperability. *J. Chem. Theory Comput.* **2017**, *13*, 3185–3197.

(S25) Smith, D. G.; Burns, L. A.; Simmonett, A. C.; Parrish, R. M.; Schieber, M. C.; Galvelis, R.; Kraus, P.; Kruse, H.; Di Remigio, R.; Alenaizan, A.; James, A. M.; Lehtola, S.; Misiewicz, J. P.; Scheurer, M.; Shaw, R. A.; Schriber, J. B.; Xie, Y.; Glick, Z. L.; Sirianni, D. A.; O'Brien, J. S.; Waldrop, J. M.; Kumar, A.; Hohenstein, E. G.; Pritchard, B. P.; Brooks, B. R.; Schaefer, H. F.; Sokolov, A. Y.; Patkowski, K.; Deprince, A. E.; Bozkaya, U.; King, R. A.; Evangelista, F. A.; Turney, J. M.; Crawford, T. D.; Sherrill, C. D. PSI4 1.4: Open-source software for high-throughput quantum chemistry. *J. Chem. Phys.* **2020**, *152*, 184108.

(S26) Turney, J. M.; Simmonett, A. C.; Parrish, R. M.; Hohenstein, E. G.; Evangelista, F. A.; Fermann, J. T.; Mintz, B. J.; Burns, L. A.; Wilke, J. J.; Abrams, M. L.; Russ, N. J.; Leininger, M. L.; Janssen, C. L.; Seidl, E. T.; Allen, W. D.; Schaefer, H. F.; King, R. A.; Valeev, E. F.; Sherrill, C. D.; Crawford, T. D. Psi4: an open-source ab initio electronic structure program. *Wiley Interdiscip. Rev.:Comput. Mol. Sci.* **2012**, *2*, 556–565.

(S27) Grüning, M.; Gritsenko, O. V.; Van Gisbergen, S. J. A.; Baerends, E. J. Shape corrections to exchange-correlation potentials by gradient-regulated seamless connection of model potentials for inner and outer region. *J. Chem. Phys.* **2001**, *114*, 652.

(S28) Lillestolen, T. C.; Wheatley, R. J. Atomic charge densities generated using an iterative stockholder procedure. *J. Chem. Phys.* **2009**, *131*, 144101.

(S29) Kendall, R. A.; Dunning, T. H.; Harrison, R. J. Electron affinities of the first-row atoms revisited. Systematic basis sets and wave functions. *J. Chem. Phys.* **1992**, *96*, 6796.

(S30) Woon, D. E.; Dunning, T. H. Gaussian basis sets for use in correlated molecular

calculations. IV. Calculation of static electrical response properties. *J. Chem. Phys.* **1994**, *100*, 2975–2988.

(S31) Weigend, F. A fully direct RI-HF algorithm: Implementation, optimised auxiliary basis sets, demonstration of accuracy and efficiency. *Phys. Chem. Chem. Phys.* **2002**, *4*, 4285–4291.

(S32) Stone, A. J.; Dullweber, A.; Engkvist, O.; Fraschini, E.; Hodges, M. P.; Meredith, A. W.; Nutt, D. R.; Popelier, P. L. A.; Wales, D. J. Orient: a program for studying interactions between molecules, version 5.0. 2018; http://www-stone.ch.cam.ac.uk/programs.html#Orient.

(S33) Virtanen, P.; Gommers, R.; Oliphant, T. E.; Haberland, M.; Reddy, T.; Cournapeau, D.; Burovski, E.; Peterson, P.; Weckesser, W.; Bright, J.; van der Walt, S. J.; Brett, M.; Wilson, J.; Millman, K. J.; Mayorov, N.; Nelson, A. R. J.; Jones, E.; Kern, R.; Larson, E.; Carey, C. J.; Polat, İ.; Feng, Y.; Moore, E. W.; VanderPlas, J.; Laxalde, D.; Perktold, J.; Cimrman, R.; Henriksen, I.; Quintero, E. A.; Harris, C. R.; Archibald, A. M.; Ribeiro, A. H.; Pedregosa, F.; van Mulbregt, P.; SciPy 1.0 Contributors, SciPy 1.0: Fundamental Algorithms for Scientific Computing in Python. *Nat. Methods* **2020**, *17*, 261–272.

(S34) Harris, C. R.; Millman, K. J.; van der Walt, S. J.; Gommers, R.; Virtanen, P.; Cournapeau, D.; Wieser, E.; Taylor, J.; Berg, S.; Smith, N. J.; Kern, R.; Picus, M.; Hoyer, S.; van Kerkwijk, M. H.; Brett, M.; Haldane, A.; del Río, J. F.; Wiebe, M.; Peterson, P.; Gérard-Marchant, P.; Sheppard, K.; Reddy, T.; Weckesser, W.; Abbasi, H.; Gohlke, C.; Oliphant, T. E. Array programming with NumPy. *Nature* **2020**, *585*, 357–362.

(S35) Clark, S. J.; Segall, M. D.; Pickard, C. J.; Hasnip, P. J.; Probert, M. J.; Refson, K.; Payne, M. First principles methods using CASTEP. *Z. Kristall.* **2005**, *220*, 567–570.

(S36) Kohn, W.; Sham, L. J. Self-Consistent Equations Including Exchange and Correlation Effects. *Phys. Rev.* **1965**, *140*, A1133.

(S37) Burke, K.; Perdew, J. P.; Wang, Y. Derivation of a Generalized Gradient Approximation: The PW91 Density Functional. In *Electronic Density Functional Theory: Recent Progress and New Directions*; Dobson, J. F., Vignale, G., Das, M. P., Eds.; Springer US: Boston, MA, 1998; pp 81–111.

(S38) Perdew, J. P.; Burke, K.; Ernzerhof, M. Generalized Gradient Approximation Made Simple. *Phys. Rev. Lett.* **1996**, *77*, 3865.

(S39) Perdew, J. P.; Burke, K.; Ernzerhof, M. Erratum: Generalized gradient approximation made simple (Phys. Rev. Lett.(1996) 77 (3865)). *Phys. Rev. Lett.* **1997**, *78*, 1396.

(S40) Hamann, D. R.; Schluter, M.; Chiang, C. Norm-Conserving Pseudopotentials. *Phys. Rev. Lett.* **1979**, *43*, 1494–1497.

(S41) Vanderbilt, D. Soft self-consistent pseudopotentials in a generalized eigenvalue formalism. *Phys. Rev. B* **1990**, *41*, 7892.
